# Cost and burden of informal caregiving of dependent older people in a rural Indian community

**DOI:** 10.1186/1472-6963-14-207

**Published:** 2014-05-07

**Authors:** Ethel M Brinda, Anto P Rajkumar, Ulrika Enemark, Jørn Attermann, KS Jacob

**Affiliations:** 1 Department of Psychiatry, Christian Medical College, Vellore 632002, India; 2 Department of Public Health, Aarhus University, Aarhus 8000, Denmark; 3 Department of Biomedicine, Aarhus University, Aarhus 8000, Denmark

**Keywords:** Caregiver, Costs, Proxy good method, Activities of Daily Living

## Abstract

**Background:**

Lack of state supported care services begets the informal caregiving by family members as the mainstay of care provided to the dependent older people in many Low and Middle Income Countries (LMICs), including India. Little is known about the time spent on caregiving, its cost and the burden experienced by these informal caregivers. We aimed to estimate the costs of informal caregiving and to evaluate the nature as well as correlates of caregivers’ burden in a rural Indian community.

**Methods:**

We assessed 1000 people aged above 65 years, among whom 85 were dependent. We assessed their socioeconomic profiles, disability, health status and health expenditures. Their caregivers’ socio-demographic profiles, mental health, and the time spent on caregiving were assessed using standard instruments. Caregiver’s burden was evaluated using Zarit Burden Scale. We valued the annual informal caregiving costs using proxy good method. We employed appropriate non-parametric multivariate statistics to evaluate the correlates of caregivers’ burden.

**Results:**

Average time spent on informal caregiving was 38.6 (95% CI 35.3-41.9) hours/week. Estimated annual cost of informal caregiving using proxy good method was 119,210 US$ in this rural community. Mean total score of Zarit burden scale, measuring caregivers’ burden, was 17.9 (95% CI 15.6-20.2). Prevalence of depression among the caregivers was 10.6% (95% CI 4.1-17.1%). Cerebrovascular disease, Parkinson’s disease, higher disability, insomnia and incontinence of the dependent older people as well as the time spent on helping Activities of Daily Living and on supervision increased caregiver's burden significantly.

**Conclusions:**

Cost and burden of informal caregiving are high in this rural Indian community. Many correlates of burden, experienced by caregivers, are modifiable. We discuss potential strategies to reduce this burden in LMICs. Need for support to informal caregivers and for management of dependent older people with chronic disabling diseases by multidisciplinary community teams are highlighted.

## Background

Population ageing is a global phenomenon, which is related with increased life expectancy and transitions in disease pattern. With the steady increase in dependent older population [1], greater demand for health services and increased health care expenditures are expected to constrain the economies of many Low and Middle Income Countries (LMICs) [2]. The potential support ratio, which is defined as the number of working-age persons aged (15-64 years) per one older person aged above 65 years, is projected to fall from current nine to four working persons by 2050 [3]. Higher dependency ratios resulting from ageing of the population will demand high economic resource consumption for caregiving.

Implications of dependency, in terms of supportive care arrangements, have different contexts in high-income countries and in LMICs. High income countries support formal caregiving to dependent older people through institutional or home aided health services and social services [4]. However, the dependent older people in many LMICs rely on their family members due to the absence of social security systems and of any formal care support. Their informal caregivers continue to provide care without any financial or physical assistance from the state. Informal caregivers in high-income countries suffer high physical, psychological and financial burden [5-7]. Their quality of life and work participation deteriorate [8,9]. Although these issues are more relevant to LMICs, pertinent research in LMICs remain sparse.

Pearlin’s stress process [10] and Yate’s stress appraisal models [11] help the theoretical understanding of caregiving burden. Knowledge on determinants of caregivers’ burden is essential to plan optimal interventions to alleviate the burden [6]. Several studies have investigated psychosocial factors [12,13], functional dependency and role conflicts [14] as the determinants of caregiving burden. However, different contexts in LMICs and cultural factors make these results less generalizable to LMICs [15]. As predictors of caregiving outcomes include socio-demographic characteristics, care needs, and time spent on caregiving among Asians [16], we aimed to evaluate these factors as the correlates of caregivers’ burden in our population.

From societal and health policy perspective, it is necessary to estimate the costs of informal caregiving [6,17]. Prior studies in LMICs, have evaluated the costs of informal care for specific disease, with little focus on informal caregivers of dependent older people [18,19]. Dearth of health economic research on informal caregiving of dependent older people in LMICs has led to lack of policies supporting the needs and rights of informal caregivers. Hence, we aimed to estimate the cost of informal caregiving and to investigate the factors associated with burden among caregivers of dependent older people in a rural Indian community.

## Methods

### Study design

We employed a cross-sectional study design to evaluate our objectives. This study was a part of 10/66 Dementia Research Group population based studies [20]. The methodology employed in this study is briefly mentioned here and has been reported elsewhere in detail [21].

### Setting

Kaniyambadi block of Vellore district is in the southern Indian state of Tamil Nadu. The Department of Community Health, Christian Medical College, Vellore, operates a community health program in this block for the past six decades. It has developed a four tier monitoring and a computerized health surveillance system of the residents of Kaniyambadi block. The community health workers, who live in the villages with the local community, provide detailed health statistics [22]. They are supervised by public health nurses and by physicians.

### Recruitment of participants

Participants above the age of 60 years were first identified using a computerised list and by door-to-door survey. All consecutive older people, aged 65 years and above, who consented, were enrolled as participants. We have already reported the factors associated with dementia [21], depression [23], disability [15] and with out-of-pocket health expenditures [24] among these participants (N = 1000). The dependent older people (n = 85), who required care, and their primary caregivers (N = 85) were identified among the study participants with the following eligibility criteria: (i) the older person was functionally dependent [25] and was unable to perform at least one of the Activities of Daily Living (ADL), including bathing, dressing, grooming, toileting, walking and feeding, without external help, (ii) he/she had a primary caregiver who provided assistance for ADL and accompanied him/her to health facilities, (iii) both the older person and his/her primary caregiver were willing to participate. A part of these caregiving dyads (n = 30), who satisfied the education adjusted 10/66 diagnostic criteria for dementia, have been previously included in the 10/66 cross-national investigation of correlates of burden among caregivers of older people with dementia in LMICs [13].

### Caregivers’ assessment

The following standard instruments were used to assess the caregivers: (i) *Zarit Burden Interview (ZBI)*[26] to assess their burden due to caregiving. It comprises 22 items, rated on 0-4 Likert scales. Total scores of ZBI range between 0 and 88 and higher scores indicate higher burden; (ii) *Self-Reporting Questionnaire (SRQ)*[27] to screen for psychiatric morbidity and depression. SRQ has 20-items, which are scored 0 or 1. Total scores of five and above screen for common mental disorders [28] and a score of eight and above identifies depression in community [29]. SRQ has been used to detect common mental disorders in primary care in India [30]; (iii) *Client Service Receipt Inventory*[31,32] to elicit the information of caregivers’ occupation, their absenteeism to provide care, their unpaid care time inputs and their income from all sources; (iv) *Caregiver Activity Survey*[33] to assess their time spent on ADL and on other care activities such as communication as well as supervision; (v) a structured proforma to assess caregiver’s socio-demographic and socioeconomic characteristics. We assessed one randomly selected co-resident family member of each older person, who is not dependent (n = 915), with SRQ and recorded their socio-demographic characteristics for further comparisons.

### Care recipients’ assessment

We assessed the dependent older participants with the following: (i) World Health Organization Disability Assessment Scale II (WHODAS II) [34] to measure the disability; (ii) Community Screening Instrument for Dementia (CSID) [35]; (iii) Neuro Psychiatric Inventory Questionnaire (NPI-Q) [36]; (iv) Geriatric Mental State (GMS) [37]; (v) History and Aetiology Schedule- Dementia Diagnosis and subtype (HAS-DDS) [38]; (vi) Client Socio-demographic and Service Receipt Inventory (CSSRI) [32] to assess the time spent on caregiver accompanied travel to various health services and time spent with health care personnel; (vii) structured questionnaire to assess the sociodemographic, socioeconomic, medical history and anthropometrics. Training of research staff involved the procedures for data collection and quality control, which were in accordance with the norms for 10/66 Dementia Research Group population studies [20,21]. The Institutional Review Board of Christian Medical College, Vellore, India, approved this study.

### Valuation of informal caregiving

We initially estimated the amount of time (hours/week) spent on informal caregiving such as assistance with ADL (T_ADL_), health service utilization (T_HSU_), and other activities (T_OA_). As the revealed preference methods are preferred to evaluate informal caregiving costs [39], we valued the time spent on informal caregiving with one of the revealed preference methods, the Proxy Good method, that values time at the market wage rate of a professional substitute [39,40]. In the absence of national formal care aids, a multipurpose health worker (MPHW) seemed as a good proxy [41] and their nominal wages per hour in 2012 were 37.5 Indian Rupees (INR) (0.7 US$) [42]. Using the proxy good method, we estimated the annual cost of informal caregiving (C_IFC_) as:

$$T_{\mathrm{IFC}}=\sum \left(T_{\mathrm{ADL}}+T_{\mathrm{HSU}}+T_{\mathrm{OA}}\right)$$

$$C_{\mathrm{IFC}}=T_{\mathrm{IFC}}*\mathrm{WR}*\left(365/7\right)$$

T_IFC_ is the total time spent on informal care activities per week and WR is the regional wage rate per hour of MPHW. Secondly, we estimated the expected growth of Indian Gross Domestic Product (GDP) with the accrual of the time lost in informal caregiving. This was estimated as a product of labor productivity (in terms of GDP per hour worked) and time spent in informal caregiving (hours/year). The labor productivity per hour in India is 3.8 US$ [43]. All costs were adjusted to Indian rupee rates using the consumer price index in 2012 [44].

### Statistical analyses

We initially analyzed the study variables using descriptive statistics. We employed logistic regression models to evaluate the factors associated with dependency among the older participants. We used Spearman rank order correlation to assess the correlation between ZBI and SRQ total scores. The distribution of our dependent variable, ZBI total score, did not follow Gaussian distribution and had influential outliers. Regression diagnostics indicated that employing ordinary least squares (OLS) regressions for evaluating the correlates of caregivers’ burden would not be valid. Hence, we employed non-parametric robust regression by STATA *rreg* command to study the association between caregiver’s burden and hypothesized variables. This method is robust to outliers and non-normality of residuals. It works iteratively performing OLS regression to compute case weights based on absolute residuals and regresses again using these weights until convergence. We assessed the coefficients of determination of our robust regression models by STATA *rregfit* command. We performed all analyses using statistical software STATA 12.1.

## Results

### Caregivers’ and care recipients' characteristics

**Figure 1:**
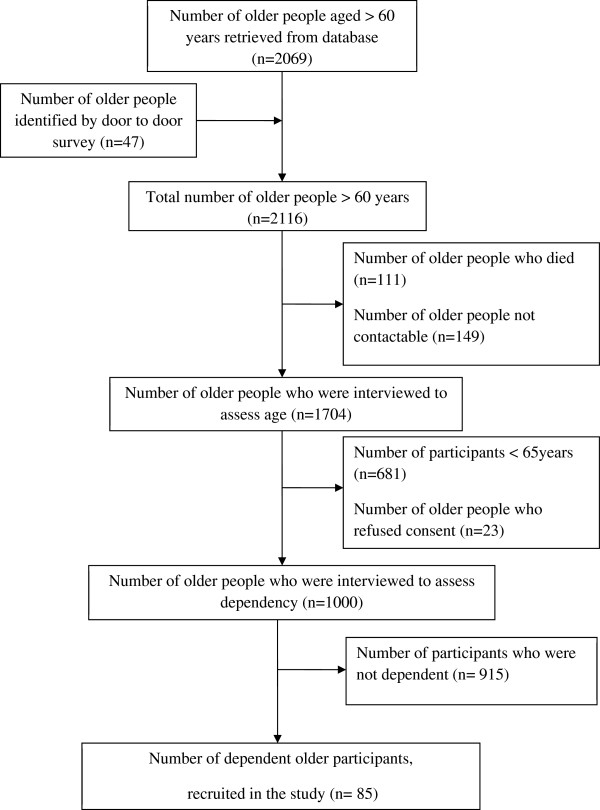
Recruitment of dependent older participants.

We present the details of recruitment of dependent older participants in Figure 1. Among 1000 older participants, the majority were women (n = 546; 54.6%) and those who lacked formal education (n = 661; 66.1%). Their (N = 1000) mean age was 72.7 (SD 5.8) years and their mean WHODAS II total score was 23.39 (SD 8.39). Further sociodemographic and clinical data of all older participants (N = 1000) have been published elsewhere [23]. We identified 85 functionally dependent older participants and included those 85 caregiving dyads in this study. Mean age of the dependent older participants (n = 85) was 74.3 (SD 6.7) years. Female gender (n = 56; OR = 1.7; 95% CI 1.1-2.7; p = 0.03), lacking formal education (n = 75; OR = 4.7; 95% CI 2.3-9.4; p < 0.001), past occupation as manual laborers (n = 78; OR = 2.5; 95% CI 1.1-5.6; p = 0.02), cerebrovascular disease (n = 8; OR = 31.6; 95% CI 8.2-121.5; p < 0.001), dementia (n = 4; OR = 11.2; 95% CI 2.7-45.8; p < 0.001), falls (n = 7; OR = 20.4; 95% CI 5.8-71.3; p p < 0.001) and incontinence (n = 13; OR = 54.9; 95% CI 15.3-197.0; p < 0.001) were significantly associated with functional dependence, while comparing the dependent older people (n = 85) with the independent older people (n = 915). Mean WHODAS II total score of dependent older participants was 35.2 (SD 13.5). We present the characteristics of their informal caregivers in Table 1. Most caregivers were women, lived with their care recipients and reported of suffering financial difficulties.

**Table 1:** Characteristics of caregivers providing informal care (n = 85)

**Caregivers' characteristics**	**n (%)**	**Mean (SD)**
Age in years		44.2 (14.1)
Women as caregiver	68 (80.0)	
No formal education	46 (54.1)	
Reported per capita monthly family income (in INR)^a^		1910.9 (1228.0)
Co-residence with the care recipient	74 (87.1)	
Reported financial difficulties due to caregiving	36 (42.4)	
Reported difficulties with social life due to caregiving	20 (23.6)	
Need for additional caregiver	54 (63.5)	
Paid caregivers	0 (0)	
Sleep disturbance secondary to caregiving	29 (34.1)	
Suicidal ideation secondary to caregiving	8 (9.4)	

^a^INR = Indian Rupees.

### Time spent on informal caregiving and its cost

The mean time spent on informal caregiving was 38.6 (SD 15.4) hours/week. We present more details on the informal caregiving time and their costs in Table 2. The estimated total annual cost of informal caregiving to these older participants (n = 85) was 119,210.2 US$. If the time, spent by these informal caregivers (n = 85) in a year, were added to the productive labor working hours, the GDP of the nation could increase by 649,902.6 US$ (3.8 US$/hour*171027 hours).

**Table 2:** Time spent on caregiving activities and cost of informal caregiving (n = 85)

**Care activities**	**Time spent on care giving mean (SD)**	**Annual costs**^**a **^**of informal caregiving mean (SD)**		**Total annual costs of informal caregiving**	
		**In Indian rupees**	**In US dollars**	**In Indian rupees**	**In US dollars**
Activities of Daily Living^b^	27.2 (8.9) (hours/week)	53139.7 (17468.9)	987.7 (324.7)	4516875.0	83956.8
Health Service Utilization^c^	17.6 (29.9) (minutes/week)	574.4 (975.4)	10.7 (18.1)	48821.3	907.4
Other activities^d^	11.1 (10.8) (hours/week)	21738.9 (21151.3)	404.1 (393.1)	1847812.5	34345.9
Total	38.6 (15.3) (hours/week)	75453.0 (30075.2)	1402.5 (559.0)	6413508.8	119210.2

^a^Proxy good method to value the informal care time close to a market price of a professional substitute; ^b^Time spent on Activities of Daily Living- includes time spent on assisting the older care recipient to eat, dress, look after their appearance, toileting and bathing; ^c^Time spent to accompany the older care recipient, while travelling to health facilities and utilizing the health services; ^d^Time spent on supervision and helping with communication of the older care recipient. Conversion value of One US dollar = 53.8 Indian rupees, according to values recorded on 25.02.2013.

### Nature of caregivers’ burden

Mean ZBI total score was 17.9 (SD 10.6). Among caregivers, 63.5% (n = 54) reported being unhappy, 21.2% (n = 18) reported lacking time for themselves, and 20.1% (n = 17) reported losing of control of their own lives due to their caregiving activities. We identified 40 (47.1%) caregivers, who were at risk for common mental disorders. Prevalence of depression among these caregivers was 10.6% (95% CI 4.1-17.1%). Total SRQ scores (Median = 4; IQR = 3) among caregivers of dependent older people (n = 85) were significantly higher (Mann-Whitney U = 30924.0; p = 0.002) than the SRQ scores (Median = 3; IQR = 3) of the family members of other older participants, who were not dependent (n = 915). After adjusting for the effects of age, gender and education, the informal caregiving role significantly worsened mental health, as evidenced by higher SRQ total scores (β = 0.8; 95% CI = 0.3- 1.3; p = 0.002). Total ZBI scores and SRQ total scores were correlated significantly among caregiving women (Spearman’s ρ = 0.2, p = 0.04). While adjusting for the effects of caregivers’ gender, higher burden due to caregiving led to significantly worse mental health, as evidenced by higher SRQ total scores (β = 0.04; 95% CI = 0.0006-0.08; p = 0.04).

### Correlates of caregivers’ burden

We present both bivariate and multivariate analyses for the correlates of caregivers’ burden in Table 3. Cerebrovascular disease, Parkinson’s disease, higher disability, urinary incontinence, insomnia, and longer hours of informal caregiving significantly worsened caregivers’ burden. A multivariate model including these significant correlates could account for 32% of the variability among the burden experienced by caregivers (R^2^ = 0.32).

**Table 3:** Factors associated with caregivers’ burden (n = 85)

**Explanatory variables**	**β**^**a **^**(95% CI)**	**p value**	**β**^**b **^**(95% CI)**	**p value**
**Care recipients' characteristics:**				
Age in years	-0.1 (-0.5; +0.2)	0.41	-0.1 (-0.4; +0.2)	0.45
Male gender	+4.3 (-0.1; +8.8)	0.06	+3.6 (-1.2; +8.5)	0.14
Household size	-0.3 (-1.1; +0.5)	0.47	-0.2 (-1.0; +0.6)	0.63
Receives pension support	-4.5 (-9.5; +0.6)	0.08	-4.0 (-9.3; +1.2)	0.13
**Sleep disturbance**	**+4.8 (+0.5; +9.1)**	**0.03**	**+5.5 (+1.3; +9.8)**	**0.01**
Had experienced fall more than once	+2.7 (-5.1; +10.5)	0.49	+2.86 (-5.1; +10.8)	0.47
**Urinary Incontinence**	**+6.9 (+1.0; +12.7)**	**0.02**	**+7.8 (+1.4; +13.5)**	**0.02**
Diabetes mellitus	-0.01 (-6.0; +6.0)	0.99	-0.6 (-6.8; +5.6)	0.85
**Cerebro vascular disease**	**+8.3 (+1.4; +15.1)**	**0.02**	**+7.5 (0.4; +14.7)**	**0.04**
**Parkinson’s disease**^**c**^	**+15.3 (+4.1; +26.4)**	**0.008**	**+15.7 (+4.1; +27.5)**	**0.009**
Dementia^d^	+0.6 (-10.8; +9.6)	0.9	+0.007 (-10.3; +10.3)	0.99
**WHODAS II total score**^**e**^	**+0.2 (+0.04; +0.3)**	**0.01**	**+0.2 (+0.07; +0.4)**	**0.004**
**Caregivers' characteristics:**				
Age in years	+0.1 (-0.03; +0.2)	0.12	+0.1 (-0.004; +0.3)^f^	0.13
Women as primary caregiver	-0.6 (-6.0; +4.8)	0.83	+0.5 (-5.0; +6.1)^g^	0.84
Spouse as primary caregiver	**+6.0 (+1.1; +10.9)**	**0.02**	+5.8 (-0.6; +12.2)	0.08
Lack of formal education	+0.1 (-4.4; +4.6)	0.96	-0.6 (-5.3; +4.1)^h^	0.79
Need to cut down work	+0.06 (-0.7; +8.3)	0.87	+0.2 (-0.7; +1.1)	0.61
Availability of additional caregiver	-1.3 (-5.8; +3.2)	0.57	-1.0 (-5.7; +3.8)	0.68
**Hours/week of informal care on ADL**^**i**^	**+1.8 (0.2; +3.4)**	**0.03**	**+1.8 (+0.1; 3.5)**	**0.03**
**Hours/week spent on supervision**	**+6.7 (+2.9; +10.4)**	**0.001**	**+6.3 (+2.5; +10.1)**	**0.001**

^a^Robust regression models with Zarit caregiver burden total score as dependent variable and the explanatory variable, presented in that row, as the only independent variable; ^b^Multiple Robust regression models including Zarit caregiver burden total score as dependent variable, the explanatory variable, presented in that row, as the independent variable, and age, gender as well as education of caregivers as covariates; ^c^Reported as diagnosed with Parkinson’s disease and had symptoms of tremor, slowness of movement, difficulty in initiating any movements; ^d^Based on DSM-IV (Diagnostic and Statistical Manual of Mental Disorders, 4th edition) diagnosis; ^e^WHO Disability Assessment Scale II 12 items total score; ^f^Multiple Robust regression model including Zarit caregiver burden total score as dependent variable and age as the independent variable, that was adjusted for gender and education of caregivers as covariates; ^g^Multiple Robust regression models including Zarit caregiver burden total score as dependent variable and female gender as the independent variable, that was adjusted for age and education of caregivers as covariates; ^h^Multiple Robust regression models including Zarit caregiver burden total score as dependent variable and the lack of formal education as the independent variable, that was adjusted for age and gender as covariates ^i^Time spent in assisting the activities of daily living; Statistically significant associations with p vales < 0.05 are presented in bold.

## Discussion

Our study estimated the cost of informal caregiving and examined the correlates of caregivers’ burden in a rural Indian community. We used standard instruments to evaluate the time spent and burden experienced by informal caregivers. The possibility of recall bias was minimized by collaborating with local community health workers and by verifying available medical records. We acknowledge the following limitations. Our valuation methods led to conservative estimates. Our data included only the time spent by caregivers in assisting ADL, supervision, communication, transportation and utilization of health services. However, data on time spent on performing instrumental daily activities (IADL) and on health care activities for chronically ill older people were not available. Indirect costs due to negative health consequences of caregiving were not included. Hence, the actual cost of informal caregiving might be higher in this community. Our cross-sectional study design could not establish any causal associations.

### Cost of informal caregiving

Despite using conservative estimates, the annual cost of informal caregiving of dependent older people in one rural Indian community (n = 85) was valued as 119,210.2 US$. As there are more than five million dependent older people in India, the estimation of annual national cost on informal caregiving can be very high [45]. Formal care services for dependent older people in high-income countries, as compared to informal caregiving, were evaluated to be cost-effective [46]. They increase women’s labor participation [47]. Loss of productive yield by the caregivers, who refrained from work, can affect a country’s economy. Evaluation of cost effectiveness of caregiving services in any LMIC needs to consider the nation’s productivity and the cost incurred by the loss of working population. The labor productivity loss of Indian GDP due to the time spent in informal caregiving by these caregivers (n = 85) amounted to 649,902.6 US$. Hence, production output that can be contributed to the nation’s economy by the informal caregivers may surpass the state expenditures on formal caregiving services for dependent older people.

### Burden due to informal caregiving

Caregivers’ burden predicts the physical and mental health outcomes of dependent older people and their caregivers [6]. Informal caregiving demands substantial effort, productive time and financial resources of caregivers. With extended hours of caregiving, caregivers may develop sleep disturbance, depression, and chronic diseases [6]. Our study adds evidence that the burden of informal caregiving worsens the mental health of caregivers, especially the women. Thus, provision of informal caregiving proceeds at the expense of caregivers’ wellbeing. Declining wellbeing of caregivers can lead to poor care of the dependent older people [48]. Neglecting to address the informal caregivers’ burden may eventually lead to ineffective care of the disabled older people in LMICs.

Our findings were consistent with the known predictors of caregivers’ burden in high-income countries [10,11]. Our results confirmed that primary stressors (functional disability) and primary appraisal (informal caregiving hours) were significantly associated with caregivers’ burden. Consistent with previous studies, stroke [18], Parkinson’s disease [49], and urinary incontinence [50] increased caregivers’ burden. An earlier cross-national study in LMIC reported that the burden of caregivers of older people with dementia was significantly associated with time spent on ADL, behavioral and psychological symptoms of dementia, and incontinence [13]. Our results confirmed these associations among caregivers of all dependent older people, regardless of their cognitive impairment, with appropriate non-parametric statistical models. Cost effective formal health and social services may modify these risk factors to reduce informal caregivers’ burden and to help dependent older people.

### Why does informal caregiving prevail as a paradigm in LMICs?

Despite well-established cost effectiveness of formal care services in high-income countries [46,47] and enormous burden suffered by informal caregivers in LMICs, many LMICs continue to persist with informal caregiving as their paradigm for caring dependent older people. The following may explain this discrepancy,

1. Culturally defined norms [51,52] oblige the family members to accept the care responsibilities as filial obligation towards older people [16]. Rural communities in LMICs hold diverse cultural beliefs that clinch family members responsible for caring dependent older people [53].

2. High gender inequality and gender stereotypes in patriarchal societies enforce the caregiving roles on many women [54]. Our study confirmed that most of the primary caregivers were women and that their mental health suffered due to caregiving burden.

3. Dearth of health economic research on informal caregiving in LMICs has left the pertinent problems under-recognized and unaddressed by policy makers in many LMICs. Social welfare support and care services to the dependent older people have not been prioritized in LMICs.

4. Health services in LMICs focus on acute management of diseases, while neglecting the need for long term disability management services [55]. Existing primary health care systems are designed to manage medical diseases and to prevent further disabilities. They are ill-equipped to care for the older people, who have already been disabled or dependent [56].

5. Rural health services do not include rehabilitative and palliative services in many LMICs.

6. Social services, which act in collaboration with health services to care for the dependent older people in high-income countries, do not exist or remain dysfunctional in many LMICs.

### Potential suggestions

The universal declaration of human rights states that every human has the right for autonomy and social security [57]. Forcing the informal caregiving role on anyone, due to the absence of state sponsored formal care services, can be considered as a violation of human rights. Hence, we suggest the following,

1. Our study revealed that stroke and Parkinson’s disease increased caregivers’ burden. Multidisciplinary teams including medical professionals, social workers, physiotherapists and occupational therapists are essential for managing chronic disabling diseases in communities. Cost-effectiveness of such multidisciplinary community interventions for dependent older people has been documented [46].

2. Health services in LMICs should not stop with acute management of cerebrovascular accidents and with medical management of underlying risk factors. They should include rehabilitation and long-term care services for stroke survivors. Long-term rehabilitation services and periodic risk assessments may impede the progression of disability [58].

3. We identified that longer caregiving hours increased caregivers’ burden. Respite services need to be implemented to help the caregivers, engaged in full-time caregiving. Provision of specialized aids and equipment, such as wheel chairs, in a subsidized cost may reduce dependency and caregiving time spent on ADL.

4. Caregiving women in this study population suffered poor mental health. Periodic educational and counseling programs as well as regular screening for psychiatric disorders are suggested for those caregiving women.

5. Dependent older people suffering insomnia and urinary incontinence led to significantly higher caregivers’ burden. Direct enquiry and detailed assessment of underlying causes should be a part of primary care assessment and management. Continence services [59] and financial assistance for continence products may reduce the burden suffered by the caregivers of incontinent older people.

6. Health services in any country cannot effectively care their dependent older people without social services assistance. Developing a functional social services system should be prioritized in LMICs to care for the dependent older people and to protect the rights of their caregivers.

7. Collaboration between public sector and non-governmental organizations are essential to provide decentralized rehabilitative and palliative services to rural India.

## Conclusions

Informal caregiving may remain as a voluntary contribution, by the motivated family members. However, reluctance of Governments to provide formal caregiving alternatives continues to coerce the vulnerable family members, especially the women, to assume caregiving roles in LMICs. There is an urgent need to care for the wellbeing of dependent older people and their families. Costs of informal caregiving should be included in the economic evaluations of health interventions and cost of illness studies in LMICs.

## Competing interests

All authors declare that they have no competing interests.

## Authors’ contributions

EMB, APR and KSJ were involved in the conception of the research question. EMB developed the research question, performed the statistical analyses and drafted the manuscript. KSJ supervised the study and data collection. APR, JA, KSJ and UE contributed to data analysis, interpretation of results and manuscript revisions. All authors read and approved the final manuscript.

## Pre-publication history

The pre-publication history for this paper can be accessed here:

http://www.biomedcentral.com/1472-6963/14/207/prepub
